# A Critical Appraisal of Emergency Medicine Specialty Training and Resignation among Residents in Emergency Medicine in Turkey

**DOI:** 10.1155/2019/6197618

**Published:** 2019-02-17

**Authors:** Murat Cetin, Sercan Bicakci, Mustafa Emin Canakci, Mevlut Okan Aydin, Basak Bayram

**Affiliations:** ^1^Department of Emergency Medicine, Tekirdag State Hospital, Tekirdag, 59030, Turkey; ^2^Department of Emergency Medicine, Namık Kemal University, 59100, Tekirdag, Turkey; ^3^Department of Emergency Medicine, Eskisehir City Hospital, 26110, Eskisehir, Turkey; ^4^Department of Emergency Medicine, University of Health Sciences Bursa Yuksek İhtisas Training and Research Hospital, 16330, Bursa, Turkey; ^5^Department of Emergency Medicine, Dokuz Eylul University, 35220, İzmir, Turkey

## Abstract

**Background and Aim:**

The nonsatisfaction among emergency medicine specialty trainees is an underrated issue in Turkey. Several previous studies have evaluated the burn-out and its consequences among physicians, but there is no study conducted with specialty trainees. The aim of this study is to evaluate the reasons for resignation among emergency medicine specialty residents in Turkey.

**Method:**

A total of 41 participants, who resigned from emergency medicine residency, were contacted by phone and invited to complete an online survey that included 25 questions about personal characteristics and departmental information.

**Results:**

Most frequent reasons of resignation were violence/security concerns (63.4%), busy work environment (53.7%), and mobbing (26.8%). Participants who reported that they have resigned due to inadequate training were mostly over 30 years old (p=0.02), continued more than 6 months to EMST (p<0.001), reported that there was no regular rotation program (p=0.003) or access to full-text scientific journals (p=0.045) in their department. All participants thought that there were deficits in the training programs, and none of them declared regret for resigning. Twenty-eight participants (68.2%) continued their specialty training at a different discipline after resignation.

**Conclusion:**

Major barriers against a high-quality and sustainable emergency medicine residency are violence in emergency services, mobbing in academic or administrative bodies, and inaccessibility to scientific resources. These obstacles can only be removed by cooperation of multiple institutions in Turkey.

## 1. Introduction

Nonsatisfaction in emergency department (ED) physicians is a significant issue in many countries, which had been investigated in numerous studies [1, 2]. Current literature data suggest that working in shifts, unpredictable work environment, complex patient management, and violence in healthcare facilities are among the key factors that pave the way for physician burn-out. The major consequences of burn-out in ED are decreased work motivation, deteriorated quality of care delivery, and attrition from ED labor [3, 4]. Attrition can be defined as refraining from emergency service clinical practice. In cases of attrition, physicians may shift to passive work positions or change their department of care service. Literature data suggests that attrition rate from emergency department practice is 1%-2% per year, and these rates could increase to 50% for 5 to 10 years-period [2]. The literature on this issue is mainly based on the physicians' conditions. But, burn-out and attrition among emergency department residents is an underrated field, and to the best of our knowledge, no previous study had evaluated this critical issue in Turkey.

After first establishment of the emergency medicine specialty training (EMST) in Dokuz Eylul University in 1993, the number of academic departments that have specialty training in Turkey has reached 25 research and training hospitals (R&T) and medical faculties of 66 universities. A recent meeting of Emergency Medicine Departments' Coordination Commission has ended up with recommendations of recruiting 6 resident positions per 30.000 patients per year, maximum shift duration of 12 hours and maximum total weekly working hour of 60 hours, and presence of an emergency medicine specialist for 24 hours in every ED to maintain a more secure training environment. Nevertheless, these recommendations could not be reached in many academic settings. These unfavorable working conditions yielded increased resigning rates among emergency medicine specialty training residents, which have reached up to 50% according to the Ministry of Health of Turkey. Despite these very high rates, causes of resignation among these residents have not been evaluated previously.

Appropriate actions to increase the job satisfaction and to decrease the resignation rates of emergency medicine residents can only be taken with identification of the actual causes of this problem. Keeping this need in mind, this study aimed to evaluate the background of resignations among residents during the first two years of EMST.

## 2. Materials and Methods

Emergency medicine specialty training residency is granted on a success from Examination for Specialty in Medicine (EMST), which is done twice a year in Turkey. Graduates from medical faculties take this examination and choose desired training program from the specialty branches that graded according to their score at the examination.

In this study, we aimed to identify residents who had resigned in the first two years of training after successfully being assigned to an EMST at the 2014-2015 EMST sessions. The lists of these residents were obtained by contacting the members of the Emergency Medicine Fellow Commission of the Turkey Emergency Medicine Association. Resigned residents were contacted by telephone calls and invited to complete the study survey.

This survey included 25 questions and was designed as a web-based online questionnaire. Data collected included age, sex, marital status, ranking of the assigned training program in EMST, knowledge level about the emergency medicine specialty training prior to EMST, and the properties of the department she/he resigned from (number of the residents and academic staff, type of the department—R&T or university, presence of any designated training programs for residents, opportunities for scientific research, and rotation programs). Additionally, any presence of violence or psychological therapy during the training was also questioned. Finally, participants were requested to declare the most important three causes for their resigning.

### 2.1. Statistical Analyses

Numerical categorical variables were presented as frequency and percent. Comparisons between independent groups were done with Chi-square test. A p value lower than 0.05 was considered statistically significant. All analyses were performed with SPSS 22 (IBM Inc., Armonk, NY, USA).

## 3. Results

A total of 1193 EMST positions were opened during the 2014-2015 EMST sessions, and no assignments were done to 395 of them (33.1%). A total 41 residents were contacted for the study, who resigned after at least one month of assignment to a training position. Twenty-six of them were male (63.4%), and 27 of them were younger than 30 years (65.9%). Twenty declared that EMST was their first choice in EMST (48.8%), and 5 reported that they have been assigned to a different specialty training program in a previous EMST before EMST (12.2%). Nineteen residents have worked less than 6 months before resigning (46.3%). General characteristics of participants were summarized in Table 1.

Twenty-one participants (51.2%) reported that there were more than 20 EMST residents in the department that they resigned from. Thirty-two participants (78%) declared that there was a regular training program, and 33 (80.5%) reported that there were some deficits in the training system of their departments. Majority of the participants (n=35; 85.4%) reported that they have been exposed to verbal or physical violence during their residency. The characteristics of the training departments were presented in Table 2.

The distribution of causes of resignation was summarized in Table 3. Accordingly, most frequent reasons were violence/security concerns (63.4%), busy work environment (53.7%), inadequate training, and realizing that emergency medicine is a wrong choice (46.3% each). Mobbing was also a significant issue, of which 26.8% of participants reported mobbing from academic staff and senior residents and 19.5% reported mobbing from administrative staff.

When the participants who reported that they have resigned due to inadequate training were analyzed further, they were mostly over 30 years old (p=0.02), continued more than 6 months to EMST (p<0.001), and reported that there was no regular rotation program (p=0.003) or access to full-text scientific journals (p=0.045) in their department. All participants thought that there were deficits in the training programs, and none of them declared regret for resigning.

Twenty-eight participants (68.2%) continued their specialty training at a different discipline after resignation. These disciplines were family medicine (n=4), psychiatry (n=4), general surgery (n=3), anesthesia (n=3), internal medicine (n=3), chest surgery (n=2), obstetrics and gynecology (n=2), emergency medicine (n=1), forensic medicine (n=1), anatomy (n=1), pediatrics (n=1), public health (n=1), orthopedics (n=1), and plastic surgery (n=1).

## 4. Discussion

Emergency departments are struggling with increasing workload all over the world. Currently, the social security and healthcare coverage systems in Turkey put a significant load on emergency care services. This workload combined with some other paramedical issues like violence in health and mobbing in workplace pave the way for nonsatisfaction of the health professionals either working or training in emergency medicine discipline. In this study, we have assessed the motivators for resignation from EMST among residents and identified that most frequent factors were violence/security concerns, work overload, and inadequate academic environment.

The pressure on emergency departments is a global issue [5]. Current literature data suggest that emergency departments possess higher rates of stress factors on physicians than other medical disciplines [6, 7]. As a consequence, intense physical and psychological stress of emergency medicine discipline raise concerns about the sustainability of this career for lifetime among emergency medicine physicians [8, 9]. According to a previous report from United Kingdom, primary reasons for not seeking a career in emergency medicine are poor quality of life of physicians, and high levels of unsocial hours of consultant work [10]. Another study from UK by Royal College of Emergency Medicine (RCEM) also reported that 65% of Welsh emergency medicine consultant physicians thought their job was unsustainable as the work overload and pressure continued in their current form [11].

Most frequent reason for resignation from EMST was violence in workplace and related personal security concerns. The United States Department of Labor defines workplace violence as “any act or threat of physical violence, harassment, intimidation, or other threatening disruptive behavior that occurs at the work site” [12]. Emergency departments have the highest rates of workplace violence than other medical departments [13]. These violent attacks not only cause acute traumatic injuries or more severe consequences like death but also cause posttraumatic symptoms, which requires therapeutic interventions [14]. Our results were consistent with this body of evidence. Accordingly, 85.4% of participants were victims of any kind of workplace violence, and 43.9% of them needed psychological support for their workplace-associated psychological stress. Apparently, this is the most critical issue that should be considered by administrators or legal entities in Turkey.

Although emergency medicine physicians have a stressful working environment, attrition rates of emergency physicians are considerably low, which was reported to be 1.5% in the annual attrition rates of emergency medicine residents [15, 16]. It was reported that 5% of the residents of internal medicine and gynecology departments fail to complete their training, but this rate is even higher in surgical fields [16, 17]. In a study evaluating the residents of all surgical branches in Canada, 26.8% of surgery residents were considering to resign where the lowest rate was in ophthalmology residents and highest rate in general surgery. The most important reason for the physicians leaving their position was declared as ‘Poor work–life balance' [18]. Attrition prevalence was reported as 18% in a systematic review assessing the prevalence and causes of attrition among surgical residents. Most frequent reasons expressed were uncontrollable lifestyle and changing the specialty field [19]. In our study, however, the physicians quitting emergency medicine residency training were mostly asserting violence and safety issues.

In the emergency departments in Turkey, the ones who suffer violence most are the young and inexperienced physicians; however the rate of physicians incurring violence in our country is not very much different than the other countries [20]. Violence incidents against physicians in their work places are an important common problem all around the world. Although this is a local study including small number of physicians, it is likely to be a general problem that security worries and increasing incidences of violence in emergency departments might be an important issue for the future of emergency medicine. Excessive workload was stated as the second reason. Despite family relations and quality of life reported as the prominent reasons for resignation in the other departments, the physicians quitting emergency department in our country most frequently blame working conditions and their worries associated with training.

Another critical problem that caused resignation from emergency department was the inadequate quality of EMST programs. Particularly, participants over 30 years of age reported poor quality of education and low levels scientific activities or accessibility to resources. This may be associated with the level of experience as a physician. Passing more years in practice may increase academic expectations, and physicians may tend to resign if their expectations are unmet. Also, these participants worked more than 6 months in emergency departments, which also may be indicative of seeking for academic development rather than seeking for a temporal job position.

## 5. Conclusions

As an overall conclusion, we think that major obstacles that should be removed for improving the quality of care and education in emergency medicine are violence in emergency services, mobbing in academic or administrative bodies, and inaccessibility to scientific resources including but not limited to online journals or databases, meetings, and courses. Unfortunately, these are major issues, and many others are in the queue, like work overload, academic staff support, and financial issues. This is a big and complex issue that has many facets, and multiple institutional approach should be initiated immediately to improve the quality of emergency medicine in Turkey.

## Figures and Tables

**Table 1:** General characteristic of the participants and their assignment to EMST.

	n	%
Sex		
*Female*	26	63.4
*Male*	15	36.6
Age		
*<30 years*	27	65.9
*30-35 years*	11	26.8
*>35 years*	3	7.3
Previous fellowship		
*Yes*	5	12.2
*No*	36	87.8
Being informed about EMST before assignment		
*A little bit*	12	29.3
*No*	29	70.7
Type of training department		
*Multiple departments*	2	4.9
*R&T*	11	26.8
*University*	28	68.3
Assigned at which EMST?		
*1*^*st*^	16	39
*2*^*nd*^	11	26.8
*3*^*rd*^	5	12.2
*4*^*th*^	2	4.9
*>5*^*th*^	6	14.6
Rank of EMST among other choices		
*1*^*st*^	20	48.8
*2*^*nd*^	3	7.3
*3*^*rd*^	4	9.8
*4*^*th*^	1	2.4
*>5*^*th*^	11	26.8
Total duration at the department before resignation		
*31 days-6 months*	19	46.3
*7 months-1 year*	10	24.4
*>1 year*	12	29.3

**Table 2:** Characteristics of the department resigned.

	n	%
Number of the EMST fellows in the department		
*<5*	2	4.9
*5-10*	4	9.8
*11-20*	14	34.1
*21-30*	11	26.8
*>30*	10	24.4
Number of the emergency medicine academic staff in the department		
*2-4*	18	43.9
*>5*	23	56.1
Regular training program in the department		
*Yes*	32	78
*No*	9	22
Accessibility to the full-text scientific publications		
*Yes*	22	53.7
*No*	19	46.3
Participation to any scientific activity (meeting/course) during fellowship		
*Yes*	20	48.8
*No*	21	51.2
Regular rotation program in the department		
*Yes*	19	46.3
*No*	22	53.7
Do you think that your department had deficits in EMST?		
*Yes*	33	80.5
*No*	8	19.5
Did specialists/academic staff actively took part in patient management?		
*Yes*	13	31.7
*No*	28	68.3
Have you been exposed to any verbal or physical violence in your department?		
*Yes*	35	85.4
*No*	6	14.6
Did you take any psychological support during residency?		
*Yes*	18	43.9
*No*	22	53.7
*Unanswered*	1	2.4
What is your current occupation?		
*Working at a different specialty training program*	23	56.1
*General practitioner*	13	31.7
*Preparing to EMST*	4	9.8
*Working at another emergency department*	1	2.4

**Table 3:** Causes of resignation from EMST.

	n	%
Violence/security concerns	26	63.4
Busy work environment	22	53.7
Inadequate training	19	46.3
Realizing that emergency medicine is a wrong choice	19	46.3
Mobbing from academic staff	11	26.8
Mobbing from senior fellows	11	26.8
Mobbing from administrative staff	8	19.5
Family-related issues	4	9.8
Insufficient number of academic staff	4	9.8
Financial issues	3	7.3
Other	7	17.1

## Data Availability

Data material has been added to supplementary files (available here).

## Supplementary Materials

Supplementary Materials Survey questions.
