# Supplementary material for: A Critical Appraisal of Emergency Medicine Specialty Training and Resignation among Residents in Emergency Medicine in Turkey
Source: Emerg Med Int. 2019 Feb 17;2019:6197618. doi: 10.1155/2019/6197618 (PMC6398009; doi:10.1155/2019/6197618)
Supplement: Supplementary Materials — Survey questions. [file 6197618.f1.pdf]

A Critical Appraisal of Emergency Medicine Specialty Training and Resignation among Residents in Emergency Medicine in Turkey

Gender?

Male ☐
Female ☐

Age?

<30 year ☐
30-35 ☐
36 year and older ☐

Are you married?

Married ☐
Single ☐
Divorced ☐

What is the faculty you graduated from?

Adnan Menderes University Faculty of Medicine ☐
Afyonkarahisar Kocatepe University Faculty of Medicine ☐
Akdeniz University Faculty of Medicine ☐
Ankara University Faculty of Medicine ☐
Atatürk University Faculty of Medicine ☐
Balıkesir University Faculty of Medicine ☐
Başkent University Faculty of Medicine ☐
Bolu İzzet Baysal University Faculty of Medicine ☐
Celal Bayar University Faculty of Medicine ☐
Cumhuriyet University Faculty of Medicine ☐
Çanakkale Onsekiz Mart University Faculty of Medicine ☐
Çukurova University Faculty of Medicine ☐
Dicle University Faculty of Medicine ☐
Dokuz Eylül University Faculty of Medicine ☐
Other ☐ ________________

Have you worked as an assistant in another area of expertise prior to emergency medicine resident?

Yes ☐
No ☐

Did you know about emergency medicine before you were an resident?

Yes ☐
No ☐
A little ☐

What branch of emergency medicine did you win in Examination for Specialty in Medicine (TUS)?

1 ☐
2 ☐
3 ☐
4 ☐
5 ≥ ☐

What is your preference for emergency medicine?

1 ☐
2 ☐
3 ☐
4 ☐
5 ☐
6 ☐
7 ☐
8 ☐
9 ☐
10 ≥ ☐

What kind of clinic you worked as an emergency medicine resident?

University Hospital (State) ☐
University Hospital (Non-state) ☐
Ministry of Health Education Research Hospital ☐
I worked in multiple clinics ☐

The clinic in which you resigned is in which region of Turkey?

- Marmara ☐
- Mediterranean ☐
- Blacksea ☐
- Aegean ☐
- Central Anatolia ☐
- East ☐
- Southeast Anatolia ☐

How long did you work at the emergency medicine clinic where you resigned?

- Less than 24 hours ☐
- 1 day-7 days ☐
- 8 Days-30 days ☐
- 31 days-6 months ☐
- 7 months-1 year ☐
- More than 1 year ☐

How many emergency medicine professionals/faculty were there in the clinic you worked with?

- 0 ☐
- 1 ☐
- 2-4 ☐
- 5≥ ☐

How many residents were there at the emergency medicine clinic you worked with?

- 5< ☐
- 5-10 ☐
- 11-20 ☐
- 21-30 ☐
- 30> ☐

Did you regret after you resigned?

- Yes ☐
- No ☐

Did you receive regular training at your clinic?

Yes ☐
No ☐

Accessibility to the full-text scientific publications?

Yes ☐
No ☐

Participation to any scientific activity (meeting/course) during fellowship?

Yes ☐
No ☐

Regular rotation program in the department?

Yes ☐
No ☐

Do you think that your department had deficits in EMST?

Yes ☐
No ☐
Enough ☐

Did specialists/academic staff actively took part in patient management?

Yes ☐
No ☐

Have you been exposed to any verbal or physical violence in your department?

Yes ☐
No ☐

Did you take any psychological support during residency?

Yes ☐
No ☐

What is your current occupation?

- Working at a different specialty training program ☐
- General practitioner ☐
- Preparing to TUS ☐
- Working at another emergency department ☐
- Working at a different specialty training program ☐

İstifa sonrası bir uzmanlık alanında asistan iseniz uzmanlık alanı nedir?

- Emergency Medicine ☐
- Forensic Medicine ☐
- Family Medicine ☐
- Anatomy ☐
- Anesthesiology and Reanimation ☐
- Brain and nerve surgery ☐
- Pediatric Surgery ☐
- Child Health and Diseases ☐
- Child and Adolescent Mental Health and Diseases ☐
- Skin and Venereal Diseases ☐
- Infectious Diseases and Clinical Microbiology ☐
- Physical Medicine and Rehabilitation ☐
- General Surgery ☐
- Thoracic Surgery ☐
- Other ☐ ________________

Causes of resignation from EMST?

- Violence / security concerns ☐
- Busy work environment ☐
- Inadequate training ☐
- Realizing that emergency medicine is a wrong choice ☐
- Mobbing from academic staff ☐
- Mobbing from senior fellows ☐
- Mobbing from administrative staff ☐
- Family-related issues ☐
- Insufficient number of academic staff ☐
- Financial issues ☐
- Other ☐
